# Palliative Home Care Nurses’ Experiences of End-of-Life Patients Expressing a Wish to Die: A Qualitative Content Analysis

**DOI:** 10.1177/23333936261474967

**Published:** 2026-07-30

**Authors:** Mattias Fernberg, Lisbeth Kristiansen

**Affiliations:** 1 25548 Department of Nursing, Sophiahemmet University, Stockholm, Sweden; 2 6311 Department of Health Sciences, Mid Sweden University, Sundsvall, Sweden

**Keywords:** end-of-life care, nurses’ experiences, palliative home care, qualitative content analysis, wish to die, Sweden

## Abstract

Expressions of a wish to die among patients with advanced illness represent a complex and ethically challenging phenomenon within palliative care. Nurses working in advanced healthcare in patients’ homes are often at the forefront of responding to such expressions in a setting characterized by close relationships and prolonged engagement. The aim of this study was to examine nurses’ experiences of caring for end-of-life patients who express a wish to die. A qualitative descriptive design was adopted. Semi-structured interviews were conducted with nine registered nurses working in advanced healthcare in patients’ homes in Sweden. Data were analysed using qualitative content analysis, combining an initial deductive phase informed by the Six S model of person-centred palliative care with a subsequent inductive analysis. One overarching theme was identified: *Alleviating suffering by engaging with the patient’s lifeworld*. Nurses interpreted expressions of a wish to die primarily as communicative acts reflecting multidimensional suffering, including physical pain, anxiety, existential distress, and loss of meaning. Four generic categories described how nurses responded through symptom relief, support for meaning-making, collaboration with relatives and the interdisciplinary team, and preservation of patient autonomy. Rather than indicating a stable desire to end life, nurses understood patients’ expressions of a wish to die as manifestations of suffering that could be alleviated through person-centred, relational, and symptom-focused care. The findings highlight the central role of nurses in palliative home care and contribute nursing-specific knowledge to the international literature on end-of-life care.

## Introduction

Within palliative care, the wish to die (WTD) or wish to hasten death (WTHD) is understood as a meaningful expression of distress rather than a simple desire for death. A foundational contribution by Monforte-Royo et al. (2012) conceptualized the WTHD as a reactive response to total suffering, encompassing the physical, psychological, social, and existential dimensions common in advanced illness. Their work showed that such expressions often reflect loss of dignity, fear of future deterioration, and threats to identity, rather than a fixed intention to shorten life. Importantly, the WTHD may function as a way of communicating unmet needs or a desire for relief and control.

This view was further developed by Rodríguez-Prat et al. (2017), who demonstrated through an updated meta-ethnography that the WTHD is dynamic, contextual, and deeply embedded in patients’ relational and sociocultural environments. From a palliative care perspective, their findings highlighted that expressions of a wish to hasten death often convey ambivalence or conditional acceptance of death (“not living like this”), underscoring the importance of careful clinical interpretation and dialogue rather than literal or reductive readings.

Recent integrative work by Rodríguez-Prat et al. (2024) has consolidated these insights in an overview of reviews that synthesizes evidence from systematic reviews and primary studies. A key contribution is the proposal that WTHD exists along a continuum, from passive death wishes to desire potentially linked to action, a framework highly relevant for palliative assessment. The overview confirms consistent associations between WTHD and depression, pain, functional decline, loss of meaning, perceived burden, and diminished quality of life. Collectively, this literature supports understanding the WTD/WTHD as an indicator of multidimensional suffering and an invitation to comprehensive, compassionate palliative care responses rather than as an endpoint preference (Monforte-Royo et al., 2012; Rodríguez-Prat et al., 2017, 2024).

From a nursing perspective, end-of-life care involves complex ethical decision-making, emotional labour, and continuous interpretation of patients’ suffering. A recent systematic review by Alanazi et al. (2024) highlights that nurses frequently encounter ethical challenges related to symptom management, communication about death, and responses to patients’ expressions of distress and desires concerning the end of life. These challenges are particularly salient in palliative care contexts, where nurses play a central role in sustaining dignity, alleviating suffering, and supporting patients and their families.

In Sweden, palliative care is progressively delivered in patients’ homes through advanced healthcare at patients’ homes (AHCPH), where interdisciplinary teams provide specialised care to individuals in the last phase of life. Registered nurses play a central role in AHCPH services and are responsible for symptom management, psychosocial and existential support, care coordination, and communication with patients and their relatives. In a legal context where euthanasia and physician-assisted suicide are prohibited, nurses are frequently required to respond to patients who express a wish to die within a framework that emphasises neither hastening nor postponing death, but rather alleviating suffering and promoting quality of life.

Previous studies have explored healthcare professionals’ experiences of patients expressing a wish to die, particularly in institutional or specialised palliative care settings. However, few studies have focused on nurses working in advanced palliative home care, where the home environment, long-term relationships, and close involvement of relatives shape the care context. Given the increasing importance of home-based end-of-life care, there is a need to deepen understanding of how AHCPH nurses interpret and respond to patients’ expressions of a wish to die.

The aim of this study was therefore to examine AHCPH nurses’ experiences of caring for end-of-life patients who express a wish to die.

## Methods

### Design

A qualitative descriptive design was employed to provide a rich and straightforward description of nurses’ experiences of caring for end-of-life patients who express a wish to die (Sandelowski, 2000, 2010). The study was grounded in an interpretive epistemological stance, recognising that participants’ experiences are understood through the meanings they ascribe to them and the researchers’ interpretations of these experiences (Patton, 2015). This approach was selected to capture nurses’ experiences and interpretations of a complex and ethically sensitive phenomenon in clinical practice. An abductive qualitative content analysis was used (Elo & Kyngäs, 2008; Graneheim & Lundman, 2004).

### Sample and Setting

Participants were purposively recruited from an advanced palliative home care service in the Stockholm region, Sweden. Inclusion criteria were being a registered nurse, having at least 2 years of professional experience in AHCPH, and caring for patients who had expressed a wish to die. Nine nurses participated: six women and three men, aged 30–60 years, with 4–20 years of nursing experience.

Sample adequacy was guided by the concept of information power (Malterud et al., 2016). The study aim was narrow and clearly defined, the sample was professionally specific and homogeneous, and the interviews yielded rich and nuanced narratives. The information provided by the nine participants was of sufficient relevance, depth, and quality to address the study aim, and the quality and specificity of the data contributed to a strong informational foundation for the analysis. Based on these considerations, the sample was considered to provide sufficient information power.

### Data Collection

The interviewer was MF, a registered nurse with experience in palliative care. No prior relationship existed between interviewer and participants. Semi-structured interviews were conducted in person at the participants’ workplaces. An interview guide informed by the Six S model of person-centred palliative care (Ternestedt, 2017) was used as a sensitizing framework when developing open-ended questions about nurses’ experiences of caring for patients who express a wish to die. The Six S model comprises six dimensions considered central to person-centred palliative care: self-image, self-determination, social relationships, symptom relief, synthesis, and strategies. The model was used to support a holistic exploration of physical, psychosocial, relational, and existential aspects of patients’ experiences and care. The Six S model is a well-established framework widely used in Swedish palliative care. Interviews lasted 30–45 minutes and were audio-recorded using a digital recording device. The interview guide is provided as Supplementary File 1.

### Data Analysis

Data were analysed using abductive qualitative content analysis, combining deductive and inductive phases (Elo & Kyngäs, 2008; Graneheim & Lundman, 2004). The analysis followed the phases of preparation, organisation, and reporting. Initially, transcripts were read repeatedly to gain familiarity with the data. During the deductive phase, meaning units were explored in relation to the Six S dimensions to ensure attention to person-centred aspects of care. Subsequently, an inductive analysis was undertaken, through which codes, subcategories, generic categories, and an overarching theme were developed from the data. To enhance analytical transparency, Table 1 presents an example of the analytical process, illustrating the progression from meaning units to categories during the abductive content analysis.

**Table 1.** Examples of the Analytic Process Overarching Theme: Alleviating suffering by Engaging With the Patient’s Lifeworld

Category	Subcategory	Subcategory
Alleviating symptoms	Relieving overpowering pain	Managing death-related anxiety
Supporting the patient to find meaning	Active life and social relationships as resources	Illness acceptance and life completion
Good care through participation	Relatives’ participation creates conditions for good care	Teamwork reduces feelings of loneliness
Caring support and patient autonomy	Preserving autonomy through communication and closeness	Supporting patient self-determination

### Ethical Considerations

The study was reviewed by the Research Ethics Council at the University of Gävle, which issued an advisory ethical opinion on February 18, 2018, stating that there were no ethical objections to conducting the study. At the time the study was conducted, formal approval from a national ethical review board was not required for interview studies involving healthcare professionals and not including sensitive personal data from patients. The study involved registered nurses only and did not collect patient data or other sensitive personal information. All participants received both written and verbal information about the study and provided informed consent before participation. Participation was voluntary, and participants were informed of their right to withdraw from the study at any time without providing a reason. Confidentiality and anonymity were ensured throughout the research process, and all data were managed in accordance with established ethical principles for human research

## Author Biographies

Mattias Fernberg, MA, RN, and Specialist Nurse in Anaesthesia Care, is a Lecturer at Sophiahemmet University, Stockholm, Sweden.

**Lisbeth Kristiansen**, PhD, RN, and Specialist in Psychiatric and Mental Health Nursing, is a Professor at Mid Sweden University, Department of Health Sciences, Sundsvall, Sweden.

## Findings

The findings are presented through one overarching theme supported by four generic categories and associated subcategories. Together, these illuminate AHCPH nurses’ experiences of caring for end-of-life patients who express a wish to die.

### Overarching Theme: Alleviating suffering by Engaging With the Patient’s Lifeworld

Nurses described that patients’ expressions of a wish to die were deeply embedded in their lived experiences of suffering. By engaging with patients’ lifeworld, listening attentively, acknowledging distress, and providing person-centred care, nurses sought to alleviate suffering underlying such expressions. Table 2 below provides an overview of the categories and their sub-categories.

**Table 2.** Overview of Categories and Subcategories

Category	Subcategory
Alleviating symptoms	Relieving overpowering pain
	Managing death-related anxiety
Supporting the patient to find meaning	Active life and social relationships as resources
	Illness acceptance and life completion
Good care through participation	Relatives’ participation creates conditions for good care
	Teamwork reduces feelings of loneliness
Caring support and patient autonomy	Preserving autonomy through communication and closeness
	Supporting patient self-determination

#### Alleviating Symptoms

As part of their experiences of caring for end-of-life patients who expressed a wish to die, nurses frequently encountered individuals suffering from overwhelming physical pain and intense anxiety related to the dying process. Nurses perceived this suffering as a central driver behind patients expressed wish to die. Consequently, alleviating symptoms was regarded as a primary nursing responsibility.

#### Relieving Overpowering Pain

Informants observed that severe pain in end-of-life patients often led to a diminished will to live. They believed that pain relief was a primary strength of AHCPH nurses. However, a significant challenge was that many patients, particularly older ones, were reluctant to take painkillers due to fears of addiction or the belief that pain was an inevitable part of cancer. One informant expressed:

Often, our patients come to us through referrals from primary healthcare or hospitals, having had no prior contact with palliative care. They often believe that pain is something they must endure and that nothing can be done about it. In their desperation, they express a desire not to live if they must endure such pain.

When pain was effectively managed, patients’ desire to die often diminished, and their desire to live became more pronounced. The informants consistently asked patients who expressed a desire to die due to pain, *“Do you really want to die?”* The typical response was *“no,”* indicating a need for help to alleviate their suffering. “*Can’t you just give me a shot? You have a whole bag full. I can’t take it anymore.”* Such requests highlight the intimate and personal nature of the nurse-patient relationship, where professionalism must be balanced with empathy. Informants noted that these expressions of a desire to die were often manifestations of frustration rather than genuine suicidal intent. They perceived pain as a multifaceted concept, where physical pain could reflect psychological or existential distress, and vice versa.

#### Managing Death-Related Anxiety

Informants also noted that, in addition to being adept at pain relief, AHCPH nurses were proficient in alleviating patient anxiety. Anxiety was often a more challenging component to manage than pain and was frequently intertwined with it.

Some patients may use morphine not because of physical pain but due to severe emotional distress, which they may not recognize or admit to themselves or others.

Anxiety, like pain, was seen as a complex condition. Informants observed that expressions of a desire to die were often rooted in anxiety rather than a genuine wish to end life. Older patients found it challenging to admit feelings of anxiety due to societal norms, whereas younger patients were more open. This generational difference may influence how nurses approach conversations about suicidal ideation, with younger patients potentially benefiting from more open and psychologically informed care strategies about their mental health struggles. This generational difference made it easier to assist younger patients in managing their anxiety. Informants believed that when patients learned to manage their anxiety through medication, counselling, or other means, their desire to die often transformed into a desire to live.

#### Supporting the Patient to Find Meaning

Nurses described how patients’ ability to find meaning, develop coping strategies, or experience a sense of life completion influenced expressions of a wish to die. Informants reported that caring for patients at the end-of-life who no longer wish to live can be complex. This complexity arises from the uniqueness of each person, shaped by their distinct experiences, social backgrounds, and coping strategies. Depending on a patient’s capacity to develop life strategies, these strategies can help them find meaning in life. Through conversations, informants help patients recognize the positive aspects of life during difficult times.

##### Active Life and Social Relationships as Resources

Informants observed that patients who lead active lives and maintain good social relationships are better equipped to develop life strategies. These patients, with numerous interests and strong social connections, were often better prepared and found it easier to devise strategies that helped them focus on the positive aspects of life, despite having a terminal illness. The patients could more readily perceive a sense of continuity in life and continue living much as before their illness. Conversely, informants noted that patients with fewer interests and a more passive lifestyle, lacking in social relationships, struggled more to develop strategies and find meaning in life. Patients with pre-existing anxiety issues also face greater difficulty in this regard. One participant said:

It seems more understandable for older individuals who may not have any friends left, but it is more challenging with younger patients. I often ask the question: 'Why?' There can be issues that can be alleviated, such as pain. I would rather die than be in pain, and we in AHCPH are proficient at managing pain. It becomes more complicated if they still insist on wanting to die because they see no meaning in life. You can try to help them see the light, but some have difficulty seeing anything other than darkness.

Several informants felt that those who saw no meaning in life were the ones most likely to express a desire to die. It was the sense of hopelessness that drove them to contemplate ending their lives. From their experience, it is uncommon for patients to commit suicide, but there are suspicions that some may accumulate medications over time and take them all at once. Since their patients are not autopsied, this remains speculative, but it is understandable why someone might choose this path, especially if they feel no sense of purpose.

It can be challenging to help some individuals see anything positive, and these are often the ones who choose to end their own lives. Informants noted that while suicide is rare, it tends to occur among patients who feel a profound sense of resignation and cannot move beyond the thought of their illness and impending death.

#### Illness Acceptance and Life Completion

Informants observed that patients who had accepted their illness often exhibited a sense of life completion. These patients, who expressed a desire to die, did so without signs of frustration or anxiety. Acceptance of their condition led some patients to feel that they had lived fulfilling lives and were ready for their lives to end. This sentiment was more common among older patients, though younger patients also demonstrated acceptance of their impending death. One informant noted,

In the late stages, particularly in the final weeks of life, patients often express a desire to die due to the overwhelming difficulties they face. However, this is not about seeking to end their lives prematurely but rather an acknowledgment that they can no longer endure their suffering. They sometimes ask for assistance in dying, but they understand that we cannot provide this, and it usually does not become an issue as they are already gravely ill and often pass away shortly after expressing this wish.

The predominant theme among patients who had accepted their illness was a natural desire for death. Some informants reported that some patients had travelled to European countries where active euthanasia is legal or planned to do so to end their lives in a suicide clinic. These patients were characterized by their sense of security and lack of abandonment, seeking assistance in dying due to prolonged severe illness and a feeling of life completion.

#### Good Care Through Participation

Collaboration with relatives and interdisciplinary teamwork were experienced as essential when caring for patients expressing a wish to die. Informants emphasized that collaboration and involvement are crucial for providing adequate care and support to end-of-life patients who no longer wish to live. The involvement of loved ones was seen as a valuable resource for both patients and nurses. Participation among team members also helped reduce feelings of loneliness, allowing them to leverage each other’s strengths and support each other’s weaknesses.

#### Relatives’ Participation Creates Conditions for Good Care

Informants unanimously agreed that loved ones play a critical role in patients’ lives. They spend the most time with the patient, handling practical tasks and addressing existential questions that arise as the disease progresses. However, relatives could also pose challenges for AHCPH nurses if they did not accept the terminal nature of the patient’s illness. Informants expressed frustration when they could not reach relatives, especially when preparing for the transition to palliative care and symptom relief. One informant stated:

Discussing death or the desire to die is taboo in our Western society. It can be difficult for relatives to accept when a loved one expresses a desire to die. Relatives may feel helpless in such situations.

Building a relationship with loved ones from the first meeting and maintaining frequent visits was seen as essential for helping both patients and relatives understand the disease and its progression. Relatives could experience discomfort when their loved ones expressed a desire to die or refused to eat or drink, leading to frustration with the AHCPH nurses. Another informant noted:

Relatives can be both the best and the worst part of my job. They can make my job a nightmare or make it easier, depending on their relationship with the patient. Families with open communication find it easier to accept that life will eventually end.

Overall, informants viewed loved ones as valuable resources, crucial for addressing both practical and existential issues. Although relatives could pose challenges if they lacked insight into the disease, their support was invaluable in making difficult decisions, such as whether to continue or cease palliative treatments.

#### Teamwork Reduces Feelings of Loneliness

The informants consistently observed that when patients expressed a desire to die or sought assistance in dying, it was typically not a genuine wish to end their life but rather an expression of frustration. The informants did not find it uncomfortable to address questions about death. Some informants noted that they had ample time with patients, during which existential questions often arose in the context of practical tasks. Four informants perceived the caregiver-patient relationship as relaxed due to the home environment, which encouraged patients to open up and express their desire to die. One informant stated:

Working with this is an ego boost—patients often call us their angels and say they couldn’t cope without us. Many have good relationships with their children or other relatives but find it difficult to discuss deeper existential issues with them. We in AHCPH “fulfill that role.

However, several informants reported difficulties in handling certain situations independently, such as when relatives had conflicting wishes or when a patient expressed persistent suicidal thoughts despite conversations with the nurse. The informants emphasized the importance of teamwork, noting that any dilemmas involving patients or their relatives were addressed by the entire AHCPH team, leveraging its various specialties to find solutions. One informant remarked:

We have wise doctors, and if a situation becomes overwhelming, we immediately convene a team meeting. It might be a medical issue, such as depression or the need for increased pain relief, where the doctor’s expertise is crucial. Our doctors are excellent listeners, and just being able to discuss difficult questions with a caring doctor can change the attitudes of both patients and relatives.

Teamwork ensured that the responsible nurse never felt alone. While informants rarely had issues responding to patients expressing a desire to die, five noted that some patients or their relatives could become burdensome, making it valuable to have team members who could provide relief. For patients without close relatives or with limited family contact, three informants highlighted the social role of the AHCPH team in offering companionship.

#### Caring Support and Patient Autonomy

Preserving patient autonomy and self-determination was described as crucial for maintaining quality of life at the end of life.

#### Preserving Autonomy Through Communication and Closeness

The disease’s impact on patients’ self-image, including increased fatigue, loss of bodily functions, and dependence on others for daily activities, can lead to a diminished quality of life and a desire to die. Informants noted that initial expressions of a desire to die were often made in frustration and typically evolved into a desire to live, rarely resurfacing. Four informants stressed the importance of listening to patients and respecting their autonomy by allowing them to do as much as they could independently. As the disease progressed, changes in self-image could reignite the desire to die. When patients requested assistance in dying, it was generally accepted that nurses could not fulfil this request. Such desires usually arose in the late palliative phase, with patients often passing away shortly thereafter. At this stage, the desire to die was more about not wanting to suffer rather than frustration. One informant noted the courage required to express a desire to die, particularly among younger patients in the early palliative phase who feared the future. The informant stated:

When younger people say they’d rather kill themselves than be bedridden, it’s based on ignorance, and it saddens me. I always tell them, ‘You will live until you die.

Six informants used the phrase “live until you die,” emphasizing the importance of living fully until death. Although some patients expressed a desire to die due to an altered self-image, it was not a common occurrence among the informants.

#### Supporting Patient Self-Determination

The right of patients to self-determination emerged as a consistent theme among the informants. This right was evident throughout the care process. Some patients reported a significant decline in their quality of life due to palliative chemotherapy, to the extent that they expressed a desire to live no longer. In these instances, AHCPH nurses played a crucial role in providing support and alleviating symptoms associated with chemotherapy, such as nausea and loss of appetite. When nausea was managed, the desire to die was seldom reiterated. However, fear and scepticism regarding new chemotherapy treatments could persist, leading to doubts about continuing further chemotherapy. Five informants emphasized the importance of quality of life and their role in supporting patients’ decisions to continue or discontinue palliative chemotherapy.

One informant stated:

I often say that treatment is sometimes provided to an absurd extent, benefiting not only but also adding to the existing suffering. I do not think oncologists always consider the side effects of cytotoxic treatments. In AHCPH, we focus more on ensuring the best possible quality of life in the remaining time, and sometimes we need to ask the patient whether their decisions reflect their own wishes or are merely following the doctors’ advice.

Three informants felt that the healthcare system did not adequately listen to patients’ wishes. They observed that the health service often adhered to care protocols while neglecting patients’ desires. AHCPH and oncology units had differing perspectives on healthcare. Five informants advocated for earlier initiation of end-of-life discussions by oncologists and earlier termination of treatments. They questioned why these discussions were not held sooner. They felt that it was often left for the doctors at AHCPH to conduct these conversations, even though they believed it should be the oncologist’s responsibility. One informant reflected:

During my basic training, I was taught that nurses should act as the patient’s advocate, standing up for the patient’s wishes, whether in discussions with relatives or other healthcare facilities. We must always be responsive. If a patient asks for help to die, I don’t impose my values; I refer to the laws and explain that I cannot assist with that. However, if the patient wishes to die, I must respect their decision, as it is their body.

The informants unanimously agreed that patient self-determination is paramount and must always be considered. Four informants had encountered patients who either left or planned to go to countries where assisted suicide is legal. They felt that, despite their personal beliefs, it was not their place to moralize. The critical factor was respecting the patient’s self-determination.

## Discussion

This study examined AHCPH nurses’ experiences of caring for end-of-life patients who express a WTD. Rather than indicating a stable desire to end life, the nurses in this study interpreted patients’ expressions of a wish to die as communicative acts reflecting multidimensional suffering. Such expressions were understood as conveying physical pain, anxiety related to dying, existential distress, relational vulnerability, or loss of meaning rather than a persistent intention to hasten death. This interpretation aligns closely with contemporary conceptualizations of the wish to die in advanced illness as a dynamic, relational, and communicative phenomenon (Monforte-Royo et al., 2012; Rodríguez-Prat et al., 2017, 2024).

By approaching patients’ death wishes as expressions of suffering, the nurses were able to respond through symptom relief, relational presence, teamwork, and support for autonomy rather than focusing on the wish to die itself. In this way, AHCPH nurses acted as interpreters of suffering and facilitators of relief, highlighting a distinctly nursing-specific contribution to end-of-life care. The findings also illustrate how the Six S model supported a holistic and person-centred approach without constraining the analysis, as nurses’ experiences extended beyond predefined categories (Ternestedt, 2017).

The home-based palliative care context appears to provide particular conditions for addressing expressions of a wish to die, including continuity, time for dialogue, and close involvement of relatives. These contextual factors may partly explain why nurses often perceived that death wishes diminished when suffering was alleviated. In this study, participants expressed frustration when they were unable to reach relatives, especially when preparing for the transition to palliative care and symptom relief. This finding is consistent with previous research highlighting the importance of family involvement in end-of-life care (Alanazi et al., 2024; Andersson et al., 2010).

The findings of this study are consistent with the broader nursing literature, which describes end-of-life care as ethically and emotionally complex. Alanazi et al. (2024) emphasize that nurses’ experiences of ethical challenges in palliative care often arise from patients’ suffering, communication about death, and the responsibility to balance symptom relief with respect for patient autonomy. In the present study, nurses’ interpretations of patients’ expressions of a WTD as manifestations of multidimensional suffering further illustrate these ethical tensions within everyday clinical practice. These findings are consistent with those of Rodríguez-Prat et al. (2024).

### Methodological Considerations

The use of information power provided a transparent and theoretically grounded justification for the sample size. The study aim was narrow and focused, and the participants had specific experience of caring for end-of-life patients who expressed a wish to die, which contributed to the relevance and richness of the data. Although the sample included both female and male nurses with varying levels of clinical experience, gender-related differences were not a focus of the analysis. Furthermore, all participants were recruited from a single advanced palliative home care service, which may limit the transferability of the findings to other organisational and cultural contexts. However, the homogeneity of the sample facilitated an in-depth exploration of the phenomenon under study and supported the generation of rich descriptions grounded in nurses’ clinical experiences.

### Clinical Implications

The findings underscore the importance of supporting AHCPH nurses in recognising expressions of a wish to die as multifaceted communications of suffering. Training in symptom management, existential communication, and interdisciplinary collaboration is essential to sustain high-quality palliative home care. The findings also highlight the importance of providing nurses with opportunities for reflection and clinical supervision when caring for patients who express a wish to die. Strengthening nurses’ competence in identifying and addressing physical, psychosocial, and existential sources of suffering may contribute to more person-centred and effective palliative care.

### Conclusion

AHCPH nurses play a pivotal role in caring for end-of-life patients who express a wish to die. By engaging with patients’ lifeworlds and interpreting such expressions as manifestations of multidimensional suffering, nurses contribute to alleviating suffering and promoting a dignified and person-centred end-of-life experience. The findings suggest that expressions of a wish to die should be understood as opportunities for dialogue and assessment rather than as straightforward indications of a desire to hasten death. This study contributes nursing-specific knowledge about how person-centred, relational, and symptom-focused care can support patients at the end of life in a home care context.

## Supplemental Material

Supplemental material - Palliative Home Care Nurses’ Experiences of End-of-Life Patients Expressing a Wish to Die: A Qualitative Content Analysis Supplemental material for Palliative Home Care Nurses’ Experiences of End-of-Life Patients Expressing a Wish to Die: A Qualitative Content Analysis by Mattias Fernberg, Lisbeth Kristiansen in Global Qualitative Nursing Research
