# Supplementary material for: Palliative Home Care Nurses’ Experiences of End-of-Life Patients Expressing a Wish to Die: A Qualitative Content Analysis
Source: Glob Qual Nurs Res. 2026 Jul 30;13:23333936261474967. doi: 10.1177/23333936261474967 (PMC13424765; doi:10.1177/23333936261474967)
Supplement: Supplemental material - Palliative Home Care Nurses’ Experiences of End-of-Life Patients Expressing a Wish to Die: A Qualitative Content Analysis [file sj-pdf-1-gqn-10.1177_23333936261474967.pdf]

INTERVIEW GUIDE

What do you consider to be the challenges and rewards of working with seriously ill patients?

Can you describe how you are affected when a patient expresses a wish to no longer live?

Can you describe what preparedness you have to respond to patients who wish to receive help from you to die?

Can you describe a particular incident that you experienced as especially difficult to handle regarding a wish to die or to receive help to die?

Do you experience that it is common for patients to wish to end their lives or to receive help to die?

Have you ever been emotionally affected by a patient who no longer wished to live or who wanted help to end their life in such a way that you found it difficult to stop thinking about it?

Can you tell me more about the incident or incidents?

Have you ever experienced that a patient has taken their own life due to their severe illness?

Can you tell me more about the incident or incidents?

What role do you consider the AHCPH team to have?
